# Death and Venous Thromboembolism Analyses among Hospitalized COVID-19-Positive Patients: A Multicenter Study

**DOI:** 10.3390/jcm12247624

**Published:** 2023-12-11

**Authors:** Asma Alshahrani, Abdullah Almoahzieie, Hanan Alshareef, Buthinah B. Alammash, Sarah Alhamidi, Abdulkarim M. Meraya, Abdullah S. Alshammari, Aziza Ajlan, Alnajla Alghofaili, Abdullah Alnassar, Nada Alshahrani, Maram Aldossari, Turkiah Alkhaldi, Marwan J. Alwazzeh, Abdullah B. Almashouf, Feras A. Alkuwaiti, Shrouq Hamed Alghamdi, Ohuod Alshehri, Mostafa Ali

**Affiliations:** 1 Department of Clinical Pharmacy, Faculty of Pharmacy, King Khalid University, Abha 62529, Saudi Arabia; ph.Ohuod@gmail.com; 2 Pharmaceutical Care Division, King Faisal Specialist Hospital and Research Center, Riyadh 11211, Saudi Arabia; amohaizeie@kfshrc.edu.sa (A.A.); aajlan@kfshrc.edu.sa (A.A.); aalnassar@kfshrc.edu.sa (A.A.); 3 Clinical Pharmacy Department, College of Pharmacy, Alfaisal University, Riyadh 11533, Saudi Arabia; 4 Department of Pharmacy Practice, Faculty of Pharmacy, University of Tabuk, Tabuk 47512, Saudi Arabia; halsharef@ut.edu.sa (H.A.); ma-ali@ut.edu.sa (M.A.); 5 Department of Pharmaceutical care services, King Fahad Hospital, Ministry of Health, AL Madinah Munawara 42351, Saudi Arabia; Ph.buth@gmail.com; 6 Pharmaceutical Care Division, Security Forces Hospital, Riyadh 11481, Saudi Arabia; alhamidisarah@gmail.com; 7 Department of Clinical Pharmacy, Pharmacy Practice Research, College of Pharmacy, Jazan University, Jazan 82722, Saudi Arabia; ameraya@jazanu.edu.sa; 8 Pharmaceutical Practice Department, College of Pharmacy, Umm Al-Qura University, Makkah 24382, Saudi Arabia; asshammari@uqu.edu.sa; 9 Department of Clinical Pharmacy, King Abdullah Medical City, Makkah 24331, Saudi Arabia; 10 Department of Internal Medicine, Prince Sultan Medical City, Riyadh 12624, Saudi Arabia; nada.m.alshahrani@gmail.com; 11 Pharmaceutical Care Division, King Abdulaziz Medical City, Riyadh 11426, Saudi Arabia; 12 Department of Clinical Pharmacy, King Fahad Medical City, Riyadh 11525, Saudi Arabia; 13 Department of Internal Medicine, King Fahd Hospital of the University, Imam Abdulrahman Bin Faisal University, Dammam 31441, Saudi Arabia; 14 Department of Pharmacy, Prince Muhammad bin Abdualaziz Hospital, Ministry of Health, Riyadh 12769, Saudi Arabia; 15 Department of Clinical Pharmacy, Faculty of Pharmacy, Assiut University, Assiut 71526, Egypt

**Keywords:** COVID-19, SARS-CoV-2, venous thromboembolism, coagulation disorders

## Abstract

Background: Coagulation disorders are frequently encountered among patients infected with coronavirus disease 2019 (COVID-19), especially among admitted patients with more severe symptoms. This study aims to determine the mortality rate and incidence and risk factors for venous thromboembolism (VTE) in hospitalized patients with COVID-19. Methods: This retrospective observational cohort study was conducted from March to July 2020 using a hospital database. All adult patients (>18 years old) with laboratory-confirmed COVID-19 were included. Laboratory data and the real-time reverse transcriptase-polymerase chain reaction (rRT-PCR) for SARS-CoV-2 were obtained from medical records. The mortality rate and the incidence of VTE were established as study results. A multivariate logistic regression analysis was performed to identify predictors of thrombotic events. Results: rA total of 1024 confirmed COVID-19 patients were treated, of whom 110 (10.7%) were deceased and 58 patients (5.7%) developed VTE. Death occurred more frequently in patients older than 50 years and those admitted to the intensive care unit (ICU, 95%) and who received mechanical ventilation (62.7%). Multivariate analysis revealed that cancer patients were two times more likely to have VTE (adjusted odds ratio = 2.614; 95% CI = (1.048–6.519); *p* = 0.039). Other chronic diseases, such as diabetes, hypertension, and chronic kidney disease, were not associated with an increased risk of VTE. Conclusions: One-tenth of hospitalized COVID-19 patients were deceased, and VTE was prevalent among patients with chronic conditions, such as cancer, despite anticoagulation therapy. Healthcare professionals should closely monitor individuals with a high risk of developing VTE to prevent unwanted complications.

## 1. Introduction

The worldwide pandemic was triggered by the appearance of the new acute severe respiratory coronavirus 2 (SARS-CoV-2) in December 2019. The range of symptoms of COVID-19, caused by SARS-CoV-2, varies widely [1,2]. It can include minor respiratory tract symptoms that resolve on their own, or more severe versions characterized by worsening pneumonia, malfunction of several organs, and even death [3,4]. The reported death rates of patients with COVID-19 throughout their hospital stay have shown significant variation, which can be attributed to variables such as the presence of other underlying health conditions and the ability of the healthcare system to provide palliative care and intensive care unit (ICU) assistance. Documented in-hospital mortality rates have varied across different regions. For example, Germany, New York, and South Korea have reported rates of approximately 20–22%, while Mexico has reported a rate of 38.5% [5,6,7,8,9].

The condition is often characterized by a variety of symptoms, including acute respiratory distress syndrome (ARDS), sepsis, fever, coughing, respiratory infections, and, in extreme cases, death. Crucially, the virus can also be carried and spread by individuals who show no symptoms [10]. Upon admission to the hospital, approximately 50% of patients have at least one comorbidity associated with COVID-19. The most frequently reported comorbidities are cardiovascular diseases, gastrointestinal problems, smell loss, skin symptoms, and coagulation disorders [11,12,13]. The existence of these comorbidities greatly increases the probability of experiencing severe symptoms and negative results of the disease. Significantly, data reveal that more than 70% of patients who require intensive care unit (ICU) support are affected by comorbidities [14].

Coagulation disorders are common in patients with COVID-19, especially those who have more severe symptoms of the condition. Abundant data have shown that COVID-19 infection is an independent and contributing risk of deep vein thrombosis (DVT), pulmonary embolism (PE), and bleeding events [15]. Thorough examination of extensive cohort studies and meta-analyzes has convincingly proven that the occurrence of thrombotic problems exceeds 20% [16,17]. An extensive investigation conducted in Sweden revealed a significant increase in the risk of DVT by a factor of five (relative incidence 4.98, 95% confidence interval 4.96 to 5.01). The risk of PE showed a notable 33-fold elevation (33.05, 32.8 to 33.3), and the risk of bleeding almost doubled (1.88, 1.71 to 2.07) within 30 days following infection [15].

Multiple laboratory evaluations have shown effectiveness in identifying VTE occurrences in individuals diagnosed with COVID-19, especially those with severe respiratory symptoms. The patient cohort has a high incidence of elevated D-dimer levels and moderate thrombocytopenia as the most common laboratory abnormalities. These diagnostic markers are essential tools for recognizing and treating thrombotic problems in patients who have tested positive for COVID-19 [18,19].

Several variables can affect blood hemostasis, increasing the likelihood of thromboembolic events [20]. These factors include older age, prolonged periods of immobility, dehydration, acute inflammation, the presence of additional cardiovascular risk factors or established cardiovascular disease, a history of VTE, inherent genetic predispositions to thrombophilia, mechanical ventilation, central venous catheterization, and recent surgical interventions. Each of these factors can contribute to the impairment of the blood vessel lining, therefore increasing the likelihood of having VTE [20]. Adopting a routine of daily evaluation and the immediate start of anticoagulant prophylaxis is an efficient approach to reducing the probability of VTE in vulnerable patients. This proactive strategy is crucial to reducing the chances of thrombotic problems, especially in those who have these indicated risk factors [21,22].

During the SARS-CoV-2 epidemic, many countries reported VTE occurrences. However, there is a lack of extensive data on this condition in Saudi Arabia. This study aims to fill this knowledge gap by investigating the mortality rate among COVID-19-positive patients admitted to hospital in Saudi Arabia. In addition, it aims to evaluate the risk of VTE in this group of patients. Furthermore, the study seeks to identify and define the risk variables that contribute to the occurrence of VTE in patients diagnosed with COVID-19 in Saudi Arabia.

## 2. Methods

### 2.1. Setting and Ethical Approval

The study included the following hospitals: Prince Sultan Medical Military City in Riyadh, King Abdullah Medical City in Makkah, King Fahad Medical City in Riyadh, Prince Mohammad Bin Abdulaziz Hospital in Riyadh, King Fahad Hospital of University in Khobar, and King Faisal Specialist Hospital and Research Center in Riyadh. All facilities in the Kingdom of Saudi Arabia are tertiary care institutions that have full-time diagnostic modalities available to diagnose COVID-19 and VTE. Ethical authorization was obtained from the research ethics committee in each hospital.

### 2.2. Study Population

Eligible patients were identified using the electronic pharmacy system. All adult patients (>18 years of age) who were hospitalized and had a laboratory confirmed SARS-CoV-2 infection, regardless of whether they had been previously diagnosed with VTE, were included. The confirmed cases were determined by conducting real-time reverse transcriptase polymerase chain reaction (rRT-PCR) testing on samples taken from the nose, throat, or sputum, as determined by the World Health Organization [19]. VTE was characterized as either DVT or PE. The hospital made diagnoses based on several criteria. First, they considered clinical characteristics such as swelling, severe pain, edema, and increased warmth of the lower extremity for cases of DVT, and chest pain, breathlessness, hemoptysis, syncope, hypotension, tachycardia, and hypoxia for cases of PE. Second, they looked for hemostatic abnormalities such as sudden increases in D-dimer levels above 0.50 mg/L and high fibrinogen levels above 200 mg/dL. Lastly, some cases of DVT were confirmed by radiographic imaging using ultrasound. No standardized diagnosis of PE was documented, including CT, chest imaging, echocardiograms, or ventilation–perfusion (VQ) scans. The development of VTE was determined using the International Classification of Disease Codes (ICD-10 version) for phlebitis and thrombophlebitis, namely I80.0-I80.3, I80.8-I80.9, and I82.9.

Exclusion criteria were patients discharged before 24 h of admission and children or patients ≤18 years old.

### 2.3. Study Design and Data Collection

This retrospective observational cohort study was conducted from March 2020 to July 2020. Patient data were retrospectively evaluated, starting from the day they were admitted to the hospital until they were discharged or passed away. A standardized data collection form was used to collect clinical data. The information obtained includes demographic data such as age, sex, body mass index, medication history, and underlying comorbidities such as acute coronary syndrome, heart failure, diabetic mellitus (DM), hypertension, dyslipidemia, chronic kidney disease, hematologic disease, cancer, thyroid dysfunction, and lung or liver disease. Furthermore, vital signs, laboratory data, rRT-PCR results for SARS-CoV-2 and coagulopathy results (fibrinogen and D-dimer levels) were also obtained.

### 2.4. Outcomes

- The primary outcome of this investigation was to determine the mortality rate among individuals admitted to hospitals with a positive diagnosis of COVID-19.
- As a secondary focus, the study aimed to elucidate the prevalence of VTE among the same cohort of COVID-19-positive patients. Additionally, the study sought to identify and analyze the various risk factors associated with the development of VTE in this specific population of patients.

### 2.5. Explanatory Variables

Our hypothesis suggests that several factors, including predisposing risk factors, existing comorbidities, specific medication use, and admission to the intensive care unit (ICU), could influence the occurrence of VTE in the studied cohort. Thus, our study incorporated the following explanatory variables.

A Predisposing factors:
  (a) Age categories: 19–39, 40–49, 50–59, ≥60 years;
  (b) Sex;
  (c) Body mass index (BMI) categories: underweight/normal (below 24.9), overweight (25–29.9), obese (≥30).
B Comorbid physical conditions:
  (a) Diabetes mellitus;
  (b) Hypertension;
  (c) Dyslipidemia;
  (d) Heart failure;
  (e) Ischemic heart disease;
  (f) Chronic kidney disease or dialysis;
  (g) Thyroid dysfunction;
  (h) Hematological disorders;
  (i) Lung disease;
  (j) Liver disease;
  (k) Cancer.
C Medications:
  (a) Antiplatelet agents;
  (b) Anticoagulants;
  (c) Anti-diabetic medications;
  (d) Angiotensin-converting enzyme inhibitors (ACEIs) and angiotensin receptor blockers (ARBs);
  (e) Spironolactone;
  (f) Statins;
  (g) Diuretics;
  (h) Beta-blockers;
  (i) Calcium channel blockers;
  (j) Inhaled corticosteroids;
  (k) Levothyroxine.
D Admission to the ICU: Indicating whether the patient was admitted to the intensive care unit.

We conducted initial bivariate analyzes to assess the relationship between each of these explanatory variables and the increased risk of VTE. Subsequently, a more comprehensive multivariate analysis was employed to determine predictors significantly associated with an elevated risk of developing VTE, as described in the subsequent sections of this study.

### 2.6. Statistical Analysis

Categorical variables were compared using either the Chi-square test or Fisher’s exact test. Initial bivariate analyzes were performed to investigate potential associations between the incidence of VTE and the various explanatory variables. This exploration involved the application of both the Chi-square test and logistic regression.

Following this initial assessment, a multivariate logistic regression approach was used to evaluate multiple predictor variables associated with the occurrence of VTE. The final model encompassed the following variables: age, sex, presence of diabetes, hypertension, dyslipidemia, heart failure, ischemic heart disease, chronic kidney disease, thyroid dysfunction, hematologic disorders, and cancer. Additionally, the model incorporated the use of ACEs or ARBs, statins, beta-blockers, and levothyroxine as medications of interest.

Statistical significance was defined as *p*-values equal to or less than 0.05. The entire statistical analysis was performed using STATA 16.

## 3. Results

The study included a total of 1024 individuals who were admitted to hospital between March 2020 and July 2020 and had confirmed cases of COVID-19. Of the patients, 653 (64%) were men and the mean age was 54 years (standard deviation 16.2), with 36.8% being 60 years or older. Hypertension and diabetes were the most common comorbidities, with prevalence rates of 43.7% and 41%, respectively. The predominant medication categories were antihypertensives (46.2%), antidiabetics (20.9%), and statins (17.9%). Table 1 provides a comprehensive summary of the characteristics of the patients. Before hospital admission, a cohort of 262 patients received antithrombotic medication to address thrombotic events such as atrial fibrillation, acute myocardial infarction (MI), and episodes of VTE. Approximately 15% of the patients were hospitalized in the ICU, while 117 individuals (11.4%) received mechanical ventilation.

### 3.1. Mortality Rate

The main objective of the study was met in 110 patients, representing 10.7% of the total. Among these patients, 52 individuals (47.2%) were using antithrombotic medication. Almost 95% of the deceased were hospitalized in the ICU prior to their deaths, and 69 cases (62.9%) required mechanical ventilation. Of the patients who had VTE while admitted to the hospital, only 10 of them died. A significant majority (79%) of the deceased patients were 50 or older.

### 3.2. Development of VTE

Anticoagulation medication was initiated for 560 patients (54.7%) after admission to the hospital. Among them, 262 patients (25.6%) received a full therapeutic dose, while 298 patients (29.1%) received a prophylactic dose. Enoxaparin was the most widely used anticoagulant, accounting for 76.4% of prescriptions. Unfractionated heparin was the second most used, with a usage rate of 19.7%. Direct oral anticoagulants (DOAC) were administered in 2.3% of the cases, while warfarin was used in only 1.6% of the cases. Significantly, of the total number of patients, 58 individuals (5.7%) had VTE and seven of them developed PE.

Comparisons between hospitalized patients with and without VTE showed similarity in comorbidities, with the exception of chronic kidney disease, thyroid dysfunction, hematologic diseases, and cancer. The appearance of these disorders was significantly higher in people with VTE (*p* = 0.032, *p* = 0.002, *p* < 0.001, *p* = 0.003, respectively; refer to Table 1). Regarding the medication history, there was a higher incidence of VTE in patients who were using ACEIs or ARB, statins, and beta-blockers (*p* = 0.004, *p* = 0.047, *p* = 0.045, respectively). Furthermore, a higher proportion of individuals with VTE had higher levels of D-dimer and fibrinogen compared to those without VTE (93.8% vs. 70.6%; *p* = 0.001; and 52.6% vs. 31.9%; *p* = 0.008, respectively). Table 1 presents a detailed summary of the characteristics of individuals admitted to the hospital with COVID-19. The table is categorized according to whether they had VTE, and it includes relevant comparisons between different factors and the presence of VTE.

### 3.3. Factors Associated with VTE

Table 2 shows the results of the multivariable logistic regression analysis, presenting an adjusted odds ratios (AOR) along with their respective 95% confidence intervals (CI). Through this analysis, patients with a confirmed cancer diagnosis emerged as the sole factor significantly associated with a higher probability of developing VTE. On thorough examination, it was observed that the count of cancer patients was significantly higher in the VTE group (8 patients, 13.8%) compared to the non-VTE group (47 patients, 4.9%; *p* = 0.003). Specifically, cancer patients exhibited a twofold increase in the probability of experiencing VTE (AOR = 2.614; 95% CI = 1.048–6.519; *p* = 0.039; Table 2). Furthermore, while comorbidities such as DM, hypertension, dyslipidemia, and heart failure demonstrated a potential association with the development of VTE, these factors did not achieve statistical significance in distinguishing between the two groups based on the appearance of VTE.

An additional multivariate logistic regression model was developed to address the significant absence of data for the D-dimer (n = 155). This model included previously identified explanatory factors along with the D-dimer to assess their association with the occurrence of VTE, as shown in Table 3. Although most of the findings were consistent with the original results, a significant supplementary observation was made about the correlation between hematologic diseases and the occurrence of VTE. The investigation demonstrated a statistically significant correlation between hematological diseases and the probability of developing VTE. Patients with hematologic diseases had a significantly higher probability of developing VTE compared to individuals without these illnesses (adjusted odds ratio: 9.064; *p* = 0.006).

## 4. Discussion

This study, conducted in many centers, investigated the mortality rate and the occurrence of VTE events in patients hospitalized with COVID-19 infection. The study observed a mortality rate of 10.7% among the participants. This rate is lower than the figures reported in other in-hospital investigations [5,6,7,8]. A significant proportion of the deceased patients (62.9%) received mechanical ventilation, perhaps indicating a correlation between the severity of the disease and mortality. However, even with a mild illness, the patients experienced mortality. A comprehensive study conducted in Germany revealed that patients who had mechanical ventilation had much higher rates of mortality during their hospital stay, and 53% of ventilated patients succumbed to their condition. On the contrary, patients who did not need ventilation had a significantly lower mortality rate of 16% [5]. In November 2022, the Saudi Ministry of Health Statistics recorded a mortality rate of 14% among the 824,747 people infected with COVID-19, resulting in a total of 9,438 deaths [23].

In this study, the incidence of VTE among hospitalized patients with COVID-19 was found to be 5.7%, even when they were receiving anticoagulation medication. However, the implementation of prophylaxis and the administration of anticoagulants resulted in a decrease in the incidence of VTE to a rate lower than 6% in this particular investigation. Previous comprehensive research, conducted in Germany, revealed that extended use of oral anticoagulant medications, such as DOAC or warfarin, was independently associated with reduced death rates and a decrease in the occurrence of blood clot-related events [6]. Due to the retrospective nature of this investigation, we were unable to determine the exact occurrence of PE in hospitalized patients, as not all patients had thoracic computed tomography. Therefore, this study has underestimated the proportion of PEs. COVID-19 infection can cause the manifestation of several diseases. Certain individuals may have a pronounced pro-inflammatory condition that may be related to coagulopathy. The exact mechanism remains unclear; however, it is likely to be attributed to a widespread inflammatory process, endothelial cells, lack of mobility, and disseminated intravascular coagulation [24]. Studies have shown that there is a higher incidence of VTE among critically ill people who have elevated levels of D-dimer and have not received anticoagulant treatment [25]. According to another study, approximately 46.1% of patients experienced the development of DVT. The risk variables that were shown to be related to a higher incidence of DVT were the CURB-65 score (ranging from 3 to 5), the Padua score, and the levels of D-dimer in hospitalized patients. Furthermore, older people, as well as those with reduced lymphocyte counts, increased APTT, and elevated D-dimer levels, exhibited risk factors for VTE [26].

Our findings suggest that the incidence of thromboembolic consequences is quite modest compared to the rates reported in meta-analyses of COVID-19 infected individuals hospitalized, which ranged from 13% to 26% [17]. However, our findings align more closely with those of a multicenter study carried out in Saudi Arabia. This investigation documented a VTE incidence of 1.5% in COVID-19 patients and highlighted that all VTE episodes, except one, took place in the ICU [27].

The only significant factor identified for VTE in our study was cancer, demonstrating that cancer patients were twice as likely to experience VTE, representing 13.8% of the VTE group. This finding is consistent with the well-established understanding that cancer increases the likelihood of developing VTE. This susceptibility in cancer patients is multifaceted, involving cancer-related hypercoagulability, tumor-induced endothelial damage, the effects of chemotherapy and radiation, surgical influences, tumor compression, inflammation, paraneoplastic syndromes, and host factors that collectively increase the risk [27]. Correspondingly, the prospective observational study by Paredes-Ruiz et al. in nonhematologic cancer patients reported a 10% incidence of VTE at 90 days among COVID-19 patients, while another study comparing cancer and noncancer patients with COVID-19 showed a lower risk of VTE in cancer patients (14% vs. 18% at 28 days), consistent with our findings [28,29,30]. However, our study evidenced a higher incidence rate associated with cancer in COVID-19 patients compared to studies reporting a 7% incidence [28]. Li et al. research and other studies highlighted active cancer, recent anticancer therapy, high-risk VTE cancer subtypes and potential risk reduction through pre-admission anticoagulant/antiplatelet therapy [27,28,29]. Approximately one-third of reported deaths in COVID-19 patients were linked to VTE, particularly impacting the prognosis of cancer patients, although our study found being elderly (above 60 years) less relevant as a risk factor for VTE in multivariable logistic regression, despite 37.9% of VTE patients falling within this age group [24,26,31].

The incidence of VTE in ICU patients was 27.6%, which was higher than in non-ICU patients. However, this difference was not statistically significant in the multivariate analysis. This discovery highlights the ongoing and significant danger of VTE in hospitalized patients, even those admitted to standard hospital wards, despite the use of thromboprophylaxis. Previous studies have also shown elevated VTE occurrence rates among ICU patients with acute and severe SARS-CoV-2 infections. This discovery underscores the ongoing difficulty of the risk of VTE in hospitalized patients, underscoring the need for careful monitoring and improved preventive measures, particularly in individuals who are severely ill [16,32].

Although comorbidities such as diabetes, hypertension, dyslipidemia, and heart failure are recognized as possible variables associated with VTE, our study did not find any significant connections between these particular conditions and the incidence of VTE. Previous studies conducted during the early wave of the pandemic indicated a higher probability of negative outcomes, such as VTE, among people with preexisting health conditions and those suffering from more severe manifestations of COVID-19. However, our current investigation did not provide notable results on the correlation between these comorbidities and the development of VTE. This disparity indicates the intricate and fluctuating influence of concurrent medical illnesses on the risk of VTE among patients with COVID-19 in various research studies and groups of individuals [15].

Acknowledging the limitations in our study, including a retrospective design and a lack of international data, limits the generalizability of our findings. Our focus on hospitalized patients excluded outpatients, potentially affecting the probability of VTE due to differing health profiles. Furthermore, the data did not have enough detail, preventing a complete analysis of factors such as specific causes of death, the occurrence of PE, the frequency and duration of pre-admission use of anticoagulant and antiplatelet medications, the history of venous thromboembolism, the subtype of cancer, and bleeding status during hospitalization. Furthermore, the study did not allow the long-term risk of VTE after infection to be evaluated by follow-up with patients after discharge. Significantly, certain relevant risk variables (such as the Padua/CURB-65 score) were not consistently documented, thus restricting a thorough evaluation of the overall risk. Furthermore, the availability of laboratory data was inconsistent and the collection process did not follow standardized timing or methodology, which could possibly impact the interpretation of our findings. These limitations need careful analysis and emphasize the importance of future investigations that have more extensive datasets and strong techniques to fill these knowledge gaps.

In conclusion, the results of this investigation underscore the association between VTE and chronic physical conditions, notably cancer, among individuals affected by COVID-19. The implications of these findings suggest a critical need for healthcare professionals to maintain vigilant surveillance of patients with chronic physical diseases and concomitant COVID-19 infection. Proactive monitoring efforts seek to reduce the incidence of VTE incidents and their related sequelae, highlighting the importance of customized preventive interventions and comprehensive treatment methods for this vulnerable group of patients.

## Figures and Tables

**Table 1:** Characteristics of hospitalized adults (>18 years old) with COVID-19 with the presence of venous thromboembolism.

	Total n = 1024 (%)	No VTE n = 966 (%)	VTE n = 58 (%)	*p*-Value *
**Demographics**				
Age (years)				0.341
19–39	209/1008 (20.7)	194/950 (20.4)	15/58 (25.9)	
40–49	191/1008 (18.9)	185/950 (19.5)	6/58 (10.3)	
50–59	237/1008 (23.5)	222/950 (23.4)	15/58 (25.9)	
≥60	371/1008 (36.8)	349/950 (36.7)	22/58 (37.9)	
Missing	16	16	0	
Sex				0.970
Female	367/1020 (36)	346/962 (36)	21/58 (36.2)	
Male	653/1020 (64)	616/962 (64)	37/58 (63.8)	
Missing	4	4	0	
Body Mass Index				0.809
Underweight/Normal (Below 24.9)	131/622(21.1)	123/576 (21.4)	8/46 (17.4)	
Overweight (25–29.9)	99/622 (15.9)	91/576 (15.8)	8/46 (17.4)	
Obese (30 or above)	392/622 (63)	362/576 (62.9)	30/46 (65.2)	
Missing	402	390	12	
**Comorbidities**				
Diabetes Mellitus				
Yes	420 (41)	395 (40.9)	25 (43.1)	0.739
No	604 (59)	571 (59.1)	33 (56.9)	
Hypertension				
Yes	447 (43.7)	415 (43)	32 (55.2)	0.069
No	577 (56.3)	551 (57)	26 (44.8)	
Dyslipidemia				
Yes	55 (5.4)	50 (5.2)	5 (8.6)	0.258
No	969 (94.6)	916 (94.8)	53 (91.4)	
Heart Failure				
Yes	40 (3.9)	38 (3.9)	2 (3.4)	0.853
No	984 (96.1)	928 (96.1)	56 (96.6)	
Heart Failure Preserved				
Yes	21/120 (17.5)	15/99 (15.2)	6/21 (28.6)	0.142
No	99/120 (82.5)	84/99 (84.8)	15/21 (71.4)	
Ischemic Heart Disease				
Yes	115 (11.2)	106 (11)	9 (15.5)	0.287
No	909 (88.8)	860 (89)	49 (84.5)	
Chronic Kidney Disease				
Yes	108 (10.5)	97 (10)	11 (19)	0.032
No	916 (89.5)	869 (90)	47 (81)	
Dialysis				
Yes	19 (1.9)	18 (1.9)	1 (1.7)	0.939
No	1005 (98.1)	984 (98.1)	57 (98.3)	
Thyroid Dysfunction				
Yes	33 (3.2)	27 (2.8)	6 (10.3)	0.002
No	991 (96.8)	939 (97.2)	52 (89.7)	
Hematological Disorders				
Yes	14 (1.4)	10 (1)	4 (6.9)	<0.001
No	1010 (98.6)	956 (99)	54 (93.1)	
Lung Disease				
Yes	60 (5.9)	56 (5.8)	4 (6.9)	0.729
No	964 (94.1)	910 (94.2)	54 (93.1)	
Liver Disease				
Yes	20 (2)	20 (2.1)	0 (0)	0.268
No	1004 (98)	964 (97.9)	58 (100)	
Cancer				
Yes	55 (5.4)	47 (4.9)	8 (13.8)	0.003
No	968 (94.6)	918 (95.1)	50 (86.2)	
Smoker				
Yes	27/333 (8.1)	24/310 (7.7)	3/23 (13)	0.369
No	306/333 (91.9)	286/310 (92.3)	20/23 (87)	
ICU admission				
Yes	146 (14.3)	130 (13.5)	16 (27.6)	0.049
No	878 (85.7)	836 (86.5)	42 (72.4)	
**Medications**				
Antiplatelet				
Yes	130 (12.7)	118 (12.2)	12 (20.7)	0.326
No	894 (87.3)	848 (87.8)	46 (79.3)	
Anticoagulant prior to admission				
Yes	262 (25.6)	252 (26.1)	10 (17.2)	0.06
No	762 (74.4)	714 (73.9)	48 (82.8)	
Anticoagulant post admission				
Yes	560 (54.7)	514 (53.2)	46 (79.3)	0.000
No	464 (45.3)	452 (46.8)	12 (20.7)	
Anti-diabetics				
Yes	214 (20.9)	197 (20.4)	17 (29.3)	0.105
No	810 (79.1)	769 (79.6)	41 (70.7)	
ACEIs/ARBs				
Yes	136 (13.3)	121 (12.5)	15 (25.9)	0.004
No	888 (86.7)	845 (87.5)	43 (74.1)	
Spironolactone				
Yes	17 (1.7)	16 (1.7)	1 (1.7)	0.969
No	1007 (98.3)	950 (98.3)	57 (198.3)	
Statin				
Yes	183 (17.9)	167 (17.3)	16 (27.6)	0.047
No	841 (82.1)	799 (82.7)	42 (72.4)	
Diuretics				
Yes	69 (6.7)	65 (6.7)	4 (6.9)	0.961
No	955 (93.3)	901 (93.3)	54 (93.1)	
Beta-blockers				
Yes	126 (12.3)	114 (11.8)	12 (20.7)	0.045
No	898 (87.7)	852 (88.2)	46 (79.3)	
Calcium Channel blockers				
Yes	142 (13.9)	133 (13.8)	9 (15.5)	0.708
No	882 (86.1)	833 (86.2)	49 (84.5)	
Inhaled Corticosteroid				
Yes	62 (6.1)	59 (6.1)	3 (5.2)	0.772
No	962 (93.9)	907 (93.9)	55 (94.8)	
Levothyroxine				
Yes	28 (2.7)	22 (2.3)	6 (10.3)	<0.001
No	996 (97.3)	944 (97.7)	52 (89.7)	
**Laboratory Data**				
High D-Dimer > 0.50 mg/L				
Yes	625/869 (71.9)	580/821 (70.6)	45/48 (93.8)	0.001
No	244/869 (28.1)	241/821 (29.4)	3/48 (6.2)	
High Fibrinogen > 200 mg/dL				
Yes	212/640 (33.1)	192/602 (31.9)	20/38 (52.6)	0.008
No	428/640 (66.9)	410/602 (68.1)	18/38 (47.4)	

Note: * significant *p*-value ≤ 0.05. ACEIs: angiotensin converting enzyme inhibitors; ARBs: angiotensin receptor blockers; COVID-19: coronavirus disease 2019.

**Table 2:** Estimates of odds ratios (OR) and 95% confidence intervals (CI) of the explanatory variables associated with the presence of venous thromboembolism.

	AOR	95% CI	*p*-Value *
Age groups			
19–39	Reference		
40–49	0.406	(0.147–1.121)	0.082
50–59	0.78	(0.332–1.831)	0.567
≥60	0.543	(0.229–1.289)	0.166
Sex			
Female	Reference		
Male	1.41	(0.771–2.578)	0.265
Diabetes			
No	Reference		
Yes	0.891	(0.457–1.74)	0.736
Hypertension			
No	Reference		
Yes	1.37	(0.694–2.705)	0.365
Dyslipidemia			
No	Reference		
Yes	1.557	(0.553–4.382)	0.402
Heart Failure			
No	Reference		
Yes	0.923	(0.205–4.159)	0.917
Ischemic Heart Disease			
No	Reference		
Yes	1.19	(0.484–2.926)	0.705
Chronic Kidney Disease			
No	Reference		
Yes	1.772	(0.818–3.837)	0.147
Thyroid Dysfunction			
No	Reference		
Yes	1.872	(0.591–5.933)	0.287
Hematological Disorders			
No	Reference		
Yes	2.727	(0.71–10.477)	0.144
Cancer			
No	Reference		
Yes	2.614	(1.048–6.519)	0.039
ICU admission			
No	Reference		
Yes	1.87	(0.995–3.51)	0.052
ACEIs/ARBs			
No	Reference		
Yes	1.62	(0.683–3.842)	0.273
Statins			
No	Reference		
Yes	0.915	(0.413–2.027)	0.827
Beta-Blockers			
No	Reference		
Yes	0.752	(0.318–1.782)	0.518
Levothyroxine			
No	Reference		
Yes	2.189	(0.656–7.311)	0.203

Note: * significant *p*-value ≤ 0.05. ACEIs: angiotensin-converting enzyme inhibitors; ARBs: angiotensin receptor blockers.

**Table 3:** Adjusted odds ratios (AOR) and their 95% confidence intervals (CI) of explanatory variables from multivariate logistic regression on the presence of venous thromboembolism (N = 869).

	AOR	95% CI	*p*-Value *
Age groups			
19–39	Reference		
40–49	0.395	(0.124–1.252)	0.115
50–59	0.702	(0.27–1.821)	0.466
≥60	0.48	(0.181–1.277)	0.141
Sex			
Female	Reference		
Male	1.442	(0.725–2.868)	0.297
Diabetes			
No	Reference		
Yes	0.897	(0.441–1.825)	0.765
Hypertension			
No	Reference		
Yes	1.226	(0.593–2.534)	0.582
Dyslipidemia			
No	Reference		
Yes	1.592	(0.496–5.11)	0.435
Heart Failure			
No	Reference		
Yes	1.397	(0.287–6.803)	0.679
Ischemic Heart Disease			
No	Reference		
Yes	1.045	(0.382–2.859)	0.932
Chronic Kidney Disease			
No	Reference		
Yes	1.818	(0.785–4.206)	0.163
Thyroid Dysfunction			
No	Reference		
Yes	1.487	(0.396–5.586)	0.557
Hematological Disorders			
No	Reference		
Yes	9.064	(1.876–43.801)	0.006
Cancer			
No	Reference		
Yes	1.158	(0.294–4.569)	0.834
ICU admission			
No	Reference		
Yes	1.32	(0.475–12.19)	0.711
ACEIs/ARBs			
No	Reference		
Yes	1.553	(0.579–4.169)	0.382
Statins			
No	Reference		
Yes	1.536	(0.652–3.618)	0.327
Beta-Blockers			
No	Reference		
Yes	0.786	(0.285–2.168)	0.642
Levothyroxine			
No	Reference		
Yes	1.614	(0.39–6.685)	0.509

Note: * significant *p*-value ≤ 0.05.

## Data Availability

The datasets used and/or analyzed during the current study are available from the corresponding author on reasonable request.

## Abbreviations

ACEIs	angiotensin-converting enzyme inhibitor
ARBs	angiotensin receptor blockers
AOR	adjusted odds ratios
CI	confidence intervals
COVID-19	coronavirus disease 2019
DVT	deep venous thrombosis
DM	diabetes mellitus
DOAC	direct oral anticoagulant
ICU	intensive care unit
ICD codes	international classification of diseases codes
MI	myocardial infarction
PE	pulmonary embolism
rRT-PCR	real-time reverse transcriptase-polymerase chain reaction
SARS-CoV-2	severe acute respiratory coronavirus 2
VTE	venous thromboembolism
VQ scan	ventilation–perfusion
